# Movement Type Prediction before Its Onset Using Signals from Prefrontal Area: An Electrocorticography Study

**DOI:** 10.1155/2014/783203

**Published:** 2014-07-14

**Authors:** Seokyun Ryun, June Sic Kim, Sang Hun Lee, Sehyoon Jeong, Sung-Phil Kim, Chun Kee Chung

**Affiliations:** ^1^MEG Center, Department of Neurosurgery, Seoul National University Hospital, Seoul 110-744, Republic of Korea; ^2^Interdisciplinary Program in Neuroscience, Seoul National University College of Natural Sciences, Seoul 151-747, Republic of Korea; ^3^Department of Neurosurgery, Seoul National University College of Medicine, 103 Daehak-ro, Jongno-gu, Seoul 110-799, Republic of Korea; ^4^Sensory Organ Research Institute, Seoul National University, Seoul 151-742, Republic of Korea; ^5^School of Design and Human Engineering, Ulsan National Institute of Science and Technology, Ulsan 689-798, Republic of Korea; ^6^Department of Brain and Cognitive Sciences, Seoul National University College of Natural Sciences, Seoul 151-747, Republic of Korea

## Abstract

Power changes in specific frequency bands are typical brain responses during motor planning or preparation. Many studies have demonstrated that, in addition to the premotor, supplementary motor, and primary sensorimotor areas, the prefrontal area contributes to generating such responses. However, most brain-computer interface (BCI) studies have focused on the primary sensorimotor area and have estimated movements using postonset period brain signals. Our aim was to determine whether the prefrontal area could contribute to the prediction of voluntary movement types before movement onset. In our study, electrocorticography (ECoG) was recorded from six epilepsy patients while performing two self-paced tasks: hand grasping and elbow flexion. The prefrontal area was sufficient to allow classification of different movements through the area's premovement signals (−2.0 s to 0 s) in four subjects. The most pronounced power difference frequency band was the beta band (13–30 Hz). The movement prediction rate during single trial estimation averaged 74% across the six subjects. Our results suggest that premovement signals in the prefrontal area are useful in distinguishing different movement tasks and that the beta band is the most informative for prediction of movement type before movement onset.

## 1. Introduction

The aim of brain-computer interface (BCI) is to translate brain signals into comprehensible information useful for sending commands to the external world [1]. In particular, BCI technology is important for patients who lack control of their motor faculties; such loss can result from a variety of issues such as spinal cord injuries, amyotrophic lateral scleroses, and brainstem strokes. A BCI can improve the quality of life for such patients by enabling them to communicate with the outside world by using their brain activities [2].

The term BCI was formulated by Vidal in 1973 [3]. Over the following four decades, numerous studies attempted to improve the accuracy and reaction time performance of BCI systems [4–6]. Nevertheless, there is still a marked time delay between patient's actions and the BCI's responses. To address this issue, some authors have focused on earlier neural signals during the premovement stage [7–9].

Voluntary movement, which contains movement intention, comes into action through movement selection, planning, and preparation [10]. Two specific brain responses reflect these aspects. First, a slow negative cortical potential occurs 2 s prior to movement onset. This potential is referred to as Bereitschaftspotential (BP) or readiness potential (RP) [11]. Second, power changes in specific frequency bands appear during the same preonset period. These changes are reflected by an amplitude decrease in cortical rhythms that are disclosed in the alpha and beta ranges [12]. Though it is widely known that the supplementary motor, premotor, and primary sensorimotor areas can be generator sources of those two brain responses, some researchers report that the prefrontal area also contributes to their generation [13–16]. However, most researches regarding the prediction of motor intention or movement type have recorded early neural signals from only the central and parietal areas covering the primary sensorimotor cortex. Those areas are closely related to motor control, but the prefrontal area also contributes to generating BP and power changes in specific frequency bands [7, 8, 17].

Two brain circuits that converge on the primary motor area contribute to human voluntary action [18–20]. One is a pathway from the supplementary motor area, which receives inputs from the basal ganglia and the prefrontal area, to the primary motor area. The other is from the premotor area, which receives inputs from sensory related areas, to the primary motor area. The first circuit, including the prefrontal area, is closely related to self-paced actions as well as the motor planning or preparation. This implies that the prefrontal area may be involved in the prediction of movement types. In practice, the prefrontal area is involved in the intention to move or in the performance of willed action as evinced by several electrophysiological studies utilizing electrocorticography (ECoG), intracerebral electrodes, and neuroimaging studies using functional magnetic resonance imaging (fMRI) [18, 21–23]. On that basis, it is suggested that prefrontal area neural signals occurring during premovement stages should be considered when predicting the type of movement that is going to occur.

In order to investigate whether the prefrontal area generates useful premovement signals, high-spatial resolution and a high signal-to-noise ratio in the cortical activity signal are required because this area is close to the premotor and supplementary motor areas. Electroencephalography- (EEG-) based BCI requires numerous types of postprocessing and multichannel information processing for BCI functioning. A practical alternative, in spite of its invasiveness, is ECoG as it has higher spatial resolution, higher amplitude, greater signal-to-noise ratio, and fewer artifacts than EEG [24, 25]. Moreover, reported in other studies [17], highly accurate movement prediction can be obtained by using only a few electrodes in an ECoG-based BCI. Thus, an ECoG-based BCI approach appears to be a robust way to investigate the contribution of the prefrontal area in movement prediction before its onset time.

In this paper, we focus on the power changes in specific frequency bands to determine whether the prefrontal area generates useful information in movement prediction during the preonset period. Several researchers have indicated that high gamma oscillation in the prefrontal area might be used for predicting movement intention and motor preparation in a BCI system [26–28]. However, few studies have been performed to determine the other properties of prefrontal activity which can be useful in movement prediction. To our knowledge, movement type classification via specific frequency band power changes in the prefrontal signals during the preonset period is sparsely documented.

In this study, by using relatively simple method, we were able to classify two types of single trial ECoG signals that preceded voluntary movements. The signals were recorded from a few electrodes on the motor related and prefrontal areas. Second, we report on our investigation into whether the prefrontal area generates useful premovement signals, and we determined the frequency range that provides the most informative signals for movement prediction. Finally, we evaluate the predictive performance obtained by including prefrontal electrodes.

## 2. Materials and Methods

### 2.1. Subjects

Six patients (three females and three males, aged 25–37 years) with intractable epilepsy participated in the study. All patients underwent chronic implantation of subdural electrodes over the prefrontal area (Brodmann areas 8, 9, 10 11, 44, 45, 46, and 47), the premotor and supplementary motor areas (Brodmann area 6), and the primary sensorimotor area (Brodmann areas 1, 2, 3, and 4). The clinical profiles of each subject are presented in Table 1. Each subject underwent magnetic resonance imaging (MRI) and computed tomography (CT) before and after subdural electrode implantations. Experimentation occurred after receiving the subjects' consent forms which were approved by the Institutional Review Board of Seoul National University Hospital (IRB number H-0912-067-304).

### 2.2. Experimental Protocol and Data Acquisition

We instructed the subjects to perform self-paced hand grasping or elbow flexion with the contralateral hand or elbow side of the implantation hemisphere. Each performed the movements precisely with an interval of more than 5 s in accordance with study directions. We emphasized the importance of movement intention immediately before performing the movements and told patients not to count the number of seconds in an interval. Each session took 5 min with 2 min of rest between each session. Three task sessions were recorded for each patient except for Subject 4 who complained of sickness related to vertigo. Only two sessions were recorded for Subject 4. The number of movements per session and the interval between movements are presented in Table 2.

Each patient had between 48 and 82 subdural electrodes (Ad-tech Medical Instrument, Racine, WI, USA) implanted. The diameter of each electrode was 4 mm with an interelectrode distance of 10 mm. The brain model and implanted electrodes were reconstructed from the individual MRI and CT images by using CURRY software (version 5.0, Compumedics Neuroscan, Charlotte, NC, USA). The ECoG data were recorded by using a 128-channel digital video monitoring system (Telefactor Beehive Horizon with an AURA LTM 64- & 128-channel amplifier system, Natus Neurology, West Warwick, RI, USA) digitized at sampling rates of 200, 400, or 1600 Hz and filtered from 0.1 to 80 Hz for the 200 Hz sampling rate and from 0.1 to 100 Hz for the 400 and 1600 Hz sampling rates. The cheekbone was used as a reference site. Additionally, electromyography (EMG) was used to detect the onset of motor performance from the opponens pollicis for hand grasping and from the biceps brachii for elbow flexion. Electrooculography (EOG) using electrodes that monitor eye movement was performed concurrently. The whole experiment was video-recorded to monitor motor performance and to obtain precise definition of movement onset.

### 2.3. Signal Preprocessing

The ECoG data were analyzed by using MATLAB software (Mathworks, Natick, MA, USA). The recorded data were downsampled to 200 Hz for unification of the various sampling rates in the analysis. The ECoG channels showing abnormal signals resulting from pathology or technical problems were excluded from further analysis. Movement onset was the time when the subject was about to move her/his hand or elbow and was determined from the EMG signals. To confirm that the EMG activity is not excited during the premovement onset period, EMG onset to preonset ratios, power ratios between EMG onset periods (0 to 1 s), and EMG preonset periods (−2 to 0 s) were calculated and averaged for all trials. Evaluated ratios were 13.18 ± 5.18 (mean ± SD) dB and 21.79 ± 5.00 dB for hand grasping and elbow flexion, respectively. In addition, no significant transient EMG bursts were detected in all trials during the premovement onset periods.

### 2.4. Feature Extraction

To extract features from premovement signals, first, epoching was performed with a window of −2 s to 0 s of movement onset (EMG onset) for the first session of each ECoG data type (hand grasping and elbow flexion) for all subjects. Note that we used only the first session for feature extraction. Trials contaminated by technical and epileptic artifacts were excluded from further analyses. 22 of the 1150 trials from all subjects (2%) were discarded. A Hamming window was applied to each epoched window. A fast Fourier transform (FFT) was used to transform single trial ECoG signals in the time domain into the frequency domain for each channel. Subsequently, power spectra were computed and averaged for all trials. Power spectral density is shown in a logarithmic scale. The frequencies of interest in a spectrum were from 1 Hz to 80 Hz. A higher frequency range could not be investigated because of limited sampling frequency. To determine which electrodes showed marked difference between the two movement types, we applied specific criteria, that is, a power difference between movement types of greater than 3 dB at a specific point and with a frequency range of 4 Hz or greater. Throughout these procedures, 13 electrodes were selected from among all electrodes of all subjects (two or three electrodes per subject). To verify that the selected electrodes from among those meeting our criteria were not chosen by chance, a bootstrap method was applied. We used the same ECoG task datasets for all subjects for the random epoch sampling (epoch start times were randomly selected). The same criteria were applied to the random sampled data. This process was repeated for all electrodes, after which the number of selected electrodes was counted. Subsequently, this procedure was iterated 500 times to obtain a distribution. The estimated *P* value associated with the bootstrap procedure was <0.002.

Finally, each subject had two or three electrodes selected by our criteria (subjects 1 and 3–6, 2 electrodes; subject 2, 3 electrodes; total, 13 electrodes). Specific frequency bands showing power differences were simultaneously selected during electrode selection. Single trial ECoG signals recorded from the selected electrodes along with the pronounced power difference frequency bands were filtered by using a custom band-pass filter. The power value of each filtered signal sample was then averaged. This average was used for feature.

### 2.5. Classification

To confirm whether the extracted features represent their respective movement type on a trial-by-trial basis, we applied a linear classifier. Specifically, a linear support vector machine (SVM), which provides relatively robust classification performance [29, 30], was used. The optimized hyperplane with a maximum margin was determined from the training dataset by applying the linear SVM. To evaluate classification performance, we used fivefold cross validation [31]. All ECoG features were randomly partitioned into five subsamples. Four of the subsamples were used for training the classifier. The remaining subsample was used for estimating performance. This process was repeated five times. Finally, the averaged correct rate from all processes represented the accuracy level for evaluating classification performance. The prediction rate was then compared with the chance level.

## 3. Results

**Figure 1:**
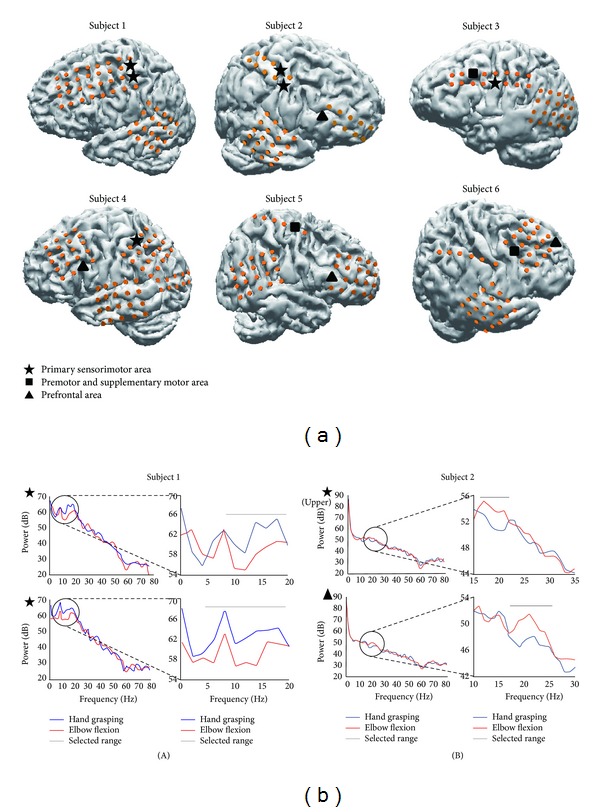
Brain models and projected electrode locations for all subjects (a). Some electrodes are not shown because they were located on invisible sites or excluded by epileptic activities. All selected electrodes of each subject are marked with an asterisk, square, and triangle on the reconstructed brain models. These three black shapes represent electrodes within the implanted areas: asterisk (primary sensorimotor area), square (premotor and supplementary motor areas), and triangle (prefrontal area). Results of FFT analysis for selected electrodes of Subject 1 (A) and Subject 2 (B) (b). The FFT results of the prefrontal area (triangle) show a distinct difference between the two movement types in the beta range. The* y*-axis has a log power scale (dB). The gray horizontal line shows a region of pronounced power differences and indicates a selected frequency band. Blue and red lines represent the hand grasping and elbow flexion movement types, respectively.

The selected electrodes from each subject were marked with three different black shapes on the respective reconstructed brain models (Figure 1(a)). Two electrodes were chosen for each subject except for Subject 2 (three electrodes chosen). The selected electrodes were placed on the primary sensorimotor area (6 electrodes in 4 subjects), premotor and supplementary areas (3 electrodes in 3 subjects), and prefrontal area (4 electrodes in 4 subjects). Notably, electrodes on the prefrontal area of four subjects were selected by applying our criteria. This suggests that the premovement neuronal activity power of the prefrontal area changes depending on the type of movement and can be detected by ECoG. The premovement power spectra for the selected electrodes for Subjects 1 and 2 are shown in Figure 1(b). The gray line indicates the frequency band that exhibits a marked power difference (>3 dB at the specific point with frequency range of ≥4 Hz) between the two movement types.

**Figure 2:**
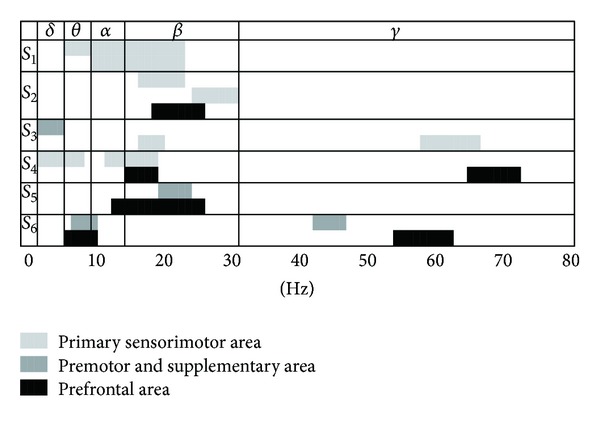
Frequency ranges of each subject's selected electrodes located in the primary sensorimotor, premotor and supplementary motor (Brodmann area 6), and prefrontal areas. The frequencies were divided into the delta, theta, alpha, beta, and gamma brain waves. The predominant frequency range was in the beta band (13–30 Hz).* S*: subject.

Subject-specific frequency bands are illustrated for all subjects in Figure 2. The results demonstrate that the filtering bands of 10 of the 13 electrodes included the beta band (13–30 Hz) and 6 of the 13 electrodes covered the alpha band (8–13 Hz). However, only 3 and 4 electrodes were used for feature extraction from the delta (<4 Hz) and gamma (30–70 Hz) bands, respectively. Furthermore, most of the selected frequency bands were in the alpha or beta ranges. In other words, the power spectral density (PSD) patterns disclosed in the alpha (8–13 Hz) and beta (13–30 Hz) rhythms were more informative than those in other bands. In particular, the beta band (13–30 Hz) was the most informative band at discriminating between the two movement types when using premovement signals, regardless of the related brain areas.

**Figure 3:**
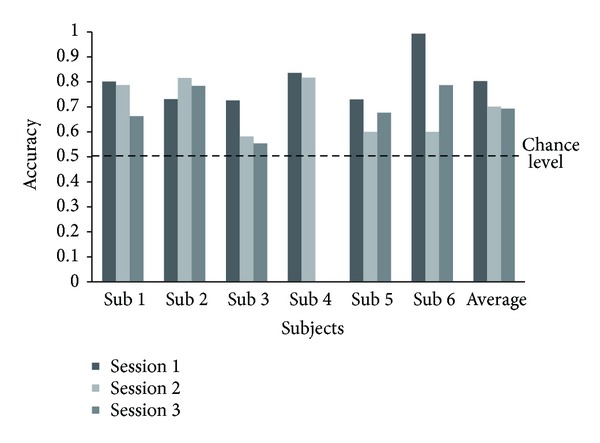
The accuracy of movement type classification. Each bar represented the accuracy of each session in each subject and the average accuracy of each session for all six subjects. The dashed line indicates the chance level for the movement classification. The average accuracy of all sessions was 74.0%.

The movement type classification accuracy across the six subjects averaged 74.0%. The average recognition rate achieved in this study ranged from 55.4 to 99.3% (Figure 3). As mentioned in Section 2.4, the subject-specific frequency bands were selected based only on the data from the first session. Subsequently, the same bands in other sessions were used to determine the reliability of our method. In other words, the subject-specific frequency bands in the first session were optimized. Thus, first session accuracy was generally higher than that in the other sessions (Figure 3). The average accuracy of the first session was 80.3% across the six subjects, well above that expected from chance. The accuracy rates of the other sessions were also significantly higher than expected from chance level. Specifically, the accuracies of the second and third sessions were 70.1% and 69.3%, respectively. These results demonstrate that the selected features provide consistent movement type classification accuracy.

**Figure 4:**
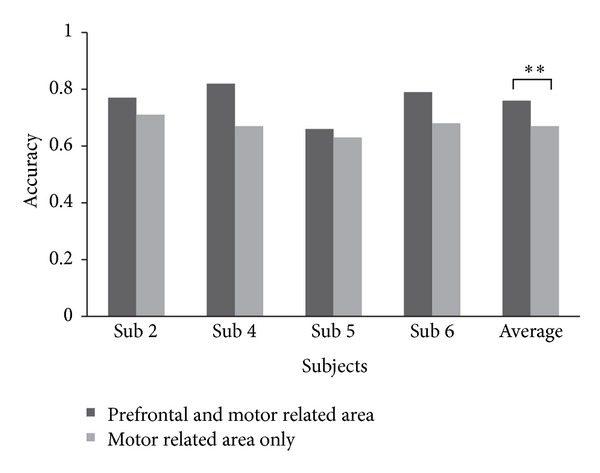
Difference in accuracy difference between two cases: a case including the prefrontal and motor related area (black, case 1) and a case including the motor related area only (gray, case 2). Each bar represents the averaged accuracy of each session in each subject and the average accuracy of each session for four subjects. The average accuracies of each case for all four subjects were 76.0 and 67%, respectively. The accuracy of case 1 was significantly higher than that of case 2 (paired *t*-test, *P* < 0.01).

To investigate whether features from the prefrontal area increase the classification accuracy, we compared the motor + prefrontal case (features from the motor related area and prefrontal area) and the motor related area only case. However, this comparison cannot be performed directly because not all subjects had features from both the prefrontal area and motor related area. Therefore, we performed this comparison for the subjects who had features from both the motor related area and prefrontal area. This result is shown in Figure 4. The accuracies of the cases that included the prefrontal and motor related area (case 1) and motor related area only (case 2) were compared in the four subjects. Classification accuracies decreased in 10 of the 11 sessions. The accuracy of case 1 was significantly higher than that of case 2 (paired *t*-test, *P* < 0.01).

## 4. Discussion

### 4.1. Regions of the Brain Involved in Prediction of Motor Planning or Preparation

Research into motor intention has predominantly focused on motor related areas activated by real motor tasks or kinesthetic illusions such as the supplementary motor area, the premotor area, and the primary sensorimotor area [32]. To this list, we added the prefrontal area as an area of interest in our study into the prediction of movement. We observed that the positions of electrodes selected by our criteria were on the primary sensorimotor area (*N* = 6), the premotor and supplementary motor area (*N* = 3), and the prefrontal area (*N* = 4). By using the signals from these areas, significant overall movement type classification accuracy (74%) was obtained. This result indicates that these regions, including the prefrontal area, contribute to prediction of motor planning or preparation. When a human performs a voluntary action, a set of decision processes within that decision determines whether to perform an action, what action to select, and whether action execution proceeds. During the decision processes, the prefrontal area, along with other regions such as the basal ganglia, supplementary motor area, premotor area, and primary sensorimotor area, is involved [18, 23]. In electrophysiological studies with intracerebral electrodes and ECoG, the prefrontal area has been observed to be a source of the slow cortical potential and frequency power shifts in the alpha and beta bands, which physiologically implies the presence of cognitive functions such as motor planning or preparatory states before movement onset [13, 14, 33]. Through our results and by considering these points, we have demonstrated that signals from the prefrontal area can be used to predict the occurrence of motor planning or preparations. In addition, our results carry important implications for paralyzed patients. In particular in patients with paralysis or trauma to the primary sensorimotor cortex, functioning of motor related areas is most likely to be damaged over time, and this damage may result in poor performance of a BCI system utilizing the primary sensorimotor area. Therefore, the usefulness of other regions needs to be investigated. In this respect, our results suggest that the prefrontal area should be considered when using a BCI to predict motor planning or preparation.

### 4.2. Importance of Beta Oscillation in Prediction of Motor Planning or Preparation

Human motor control mechanisms are associated with changes in the neuronal oscillations in motor related areas. Accordingly, several electrophysiological studies of motor preparation and execution have focused on oscillatory activity in the human cortex [8, 34, 35]. Specifically, alpha and beta band neuronal oscillatory activities during motor preparation have been quantified as exhibiting decreases or increases in power relative to a baseline period over the prefrontal, premotor and supplementary motor, and primary sensorimotor areas, and this power shift is generally referred to as an event-related desynchronization (ERD) or event-related synchronization (ERS) indicating a state of active cortical processing [14, 33]. As shown in Figure 2, beta waves (13–30 Hz) as signal features were more common than the other waves. This implies that beta band activity is the most informative for predicting movement types. This result partly supports findings in previous studies that have demonstrated that the most specific feature of premovement signals for classifying movement type is in the 8–30 Hz range [7, 8]. Several studies into brain neural oscillation have indicated that beta frequency neural oscillation might encode specific information related to motor activity or preparation [36], and it is modulated by the future task during the intention and preparation periods [37]. In addition, beta band activity can represent the status quo by receiving new information about the state or the motor command [38]. In an animal study, beta band oscillation reflected not only the maintenance of a motor plan, but also the decision outcome [39]. In contrast to beta oscillation, alpha oscillation did not fully represent the selection of the mode of action and it was not modulated by the task, but it did reflect the motor preparation state [37, 40]. To conclude, the beta frequency oscillation is an important neural activity pattern in classifying movement types.

### 4.3. Practical Advantages of Our Movement Prediction Approach Using ECoG

In this study, we showed that movement type could be predicted before the subject moves by using only three or fewer ECoG electrodes. This result has profound implications for an ECoG-based BCI system. The capacity to utilize a minimal number of electrodes in an invasive BCI system would reduce the extent of external injuries to users and would lighten their surgical burden. In addition, the feature extraction method described herein is relatively simple and does not require more complex noise reduction and signal classification methods such as those in independent component analysis and principle component analysis.

In our study, once the predictive electrodes and their associated specific frequency bands were selected from our first session data, prediction accuracy was approximately maintained during subsequent sessions. This predictive consistency indicates that an ECoG-based BCI system that utilized our approach would be helpful when implementing a robust, reliable, real-time movement classification system because of its high signal stability. In general, ECoG data obtained from the surface of the cortex has several advantages including better location stability, greater freedom from muscle and movement artifacts, higher signal-to-noise ratio, broader bandwidth, higher amplitude, and higher spatial resolution over EEG recordings [24]. Therefore, we suggest that our ECoG-based approach can provide useful preonset information about movements and is a good prospect for incorporation into a BCI system.

### 4.4. Prediction of Voluntary Movement Using Premovement Signals for Fast BCI Responses

Based on our successful prediction results, we anticipate that our method of preonset movement prediction may allow faster BCI responses. For successive prediction of a complex voluntary movement, a BCI system should perform four steps: onset prediction, movement type prediction, precise prediction of movement, and offset detection. These steps must be done sequentially. Recently, several researchers demonstrated that the onset and direction of human voluntary movement could be detected by using premovement signals of motor related areas [8, 9]. Considering these works and the results of our study, the initial two BCI steps can be performed before movement onset. In that case, a BCI system could initiate the third step faster, although postonset signals are needed for performing that step. In particular, such a BCI system could provide additional setup time for real-time prediction of complex movement such as three-dimensional trajectory estimation and bimanual movement prediction, which typically require long computation times.

### 4.5. Other Considerations and Limitations

Although we obtained high classification accuracy by using premovement signals from motor related and prefrontal areas, we did not directly compare the accuracy levels among the prefrontal, premotor and supplementary motor, and primary sensorimotor areas. Comparing the individual accuracies of each area by adding or subtracting electrodes in other areas is not effective because our classification accuracy was optimized by the extracted features. In this study, the areas for the selected electrodes were mixed, except for those in the primary sensorimotor area (Subject 1). However, we tested all electrodes of a subject to extract features by using criteria that selected specific frequency band that showed a marked power difference. In addition, the criteria did not contain* a priori* information about the location of the electrodes. In other words, the excluded electrodes did not show marked power difference between the two movement types. To compare the accuracy of each area directly, a feature extraction method that functioned without electrode selection would be required. However, such an approach was beyond the scope of this study. Hence, although there are several methodological limitations to our study, our results imply that the prefrontal area should be considered when attempting to predict motor planning or preparation because the neuronal activities in this area were shown to contribute to the classification of two movement types in four of our six subjects.

In this paper, we could not investigate the high gamma neuronal activities because of the limited sampling rates. Several researchers have indicated high gamma power changes in the dorsolateral prefrontal cortex during movement preparation and mental processing [26–28]. According to these findings, broadband high gamma power was altered depending on the movement stages which are determined by the pre- and postmovement time series. Taking into account these findings, high gamma power may be important features in movement type classification. In our study, some prefrontal area features contained gamma power in a relatively high frequency range. Although the selected frequency ranges of these features did not cover a broad range, they might reflect the previous findings. Hence, further investigation is needed to include features from high gamma neuronal activity to improve the classification accuracy.

In this study, the averaged classification accuracy was 74%. This is considered a successful result in movement prediction using the premovement stage signal. However, our classification accuracy could be improved by several ways. First, considering feature interaction might improve our classification accuracy. Although the signals of adjacent ECoG electrodes are less similar than that of the EEG electrodes, large scale neuronal oscillatory activity such as alpha oscillations in the sensorimotor area might increase the signal dependency among adjacent ECoG electrodes. Second, feature extraction taking into account time dependency during the premovement period (−2 to 0 s) might improve our BCI system. Many studies have indicated that there are several neural states during that period [11]. In addition, the electrophysiological brain signal is highly variable over time even in time-locked event-related responses. Therefore, a time-dependent feature extraction method such as short time Fourier transforms during the preonset movement period might improve the classification accuracy of our model.

## 5. Conclusion

In this paper, we demonstrate that movement type can be predicted by including prefrontal signals before the subject moves. Our results suggest that the prefrontal area can generate meaningful neuronal activity signals that can be used to predict movement before the movement occurs. Our results also suggest that beta band oscillation is the most informative for prediction of movement types before movement onset. Our findings should be of interest to those applying BCI systems in neurological rehabilitation. Our approach to ECoG-based BCI systems that utilizes signals provided by the prefrontal area carries important implications for patients with paralysis or trauma to the primary sensorimotor area.

## Figures and Tables

**Table 1:** Clinical profiles.

Subject	Age	Sex	Side of hand motion	Electrodes	
				Location	Number
Subject 1	25	Female	Right	Left hemisphere	72
Subject 2	36	Male	Left	Right hemisphere	52
Subject 3	26	Female	Right	Left hemisphere	48
Subject 4	26	Female	Right	Left hemisphere	82
Subject 5	37	Male	Left	Right hemisphere	58
Subject 6	28	Male	Left	Right hemisphere	58

**Table 2:** Behavior information. The number of movements per session and interval between movements of all subject.

Subject	Hand grasping				Elbow flexion			
	Session 1	Session 2	Session 3	Interval (s)	Session 1	Session 2	Session 3	Interval (s)
Subject 1	49	49	40	6.64 ± 1.12	42	36	40	7.75 ± 1.09
Subject 2	27	28	18	12.65 ± 3.96	25	21	19	13.84 ± 2.99
Subject 3	30	42	36	8.47 ± 1.81	32	37	29	9.41 ± 1.71
Subject 4	39	47	n.a.	7.39 ± 2.04	34	35	n.a.	9.44 ± 1.93
Subject 5	35	44	36	8.75 ± 2.21	28	31	29	11.28 ± 2.55
Subject 6	33	37	32	9.05 ± 2.68	25	28	15	12.68 ± 3.30

n.a.: not applicable.

Interval (s): mean ± SD.
